# Fabrication of κ-Carrageenan Fibers by Wet Spinning: Spinning Parameters

**DOI:** 10.3390/ma4101805

**Published:** 2011-10-11

**Authors:** Lingyan Kong, Gregory R. Ziegler

**Affiliations:** Pennsylvania State University, University Park, PA 16802-1505, USA; E-Mail: lzk119@psu.edu

**Keywords:** κ-carrageenan, fiber spinning, wet-spinning

## Abstract

This study demonstrates the fabrication of κ-carrageenan fibers by a wet-spinning method and discusses three important spinning parameters: coagulation bath composition, spinning rate and post-spinning mechanical drawing. The as-spun fiber diameter decreased with KCl and ethanol concentration in the coagulation bath. In general, the ultimate tensile stress and elongation at break both increased for KCl concentration from 0.1 to 0.5 M with and without ethanol, with no significant change above 0.5 M. Spinning rate affected the dope flow and thus the polymer orientation (apparent viscosity) and fiber morphology. At spinning rates between 0.25 mL/min and 0.33 mL/min, the fiber diameter reached a minimum and the fiber surface was smooth. Both an increase and decrease from this spinning rate range increased the fiber diameter and roughness of the fiber surface. Post-spinning drawing of the fiber resulted in even smaller fiber diameter.

## 1. Introduction

The use of petroleum-based synthetic polymers has permeated everyday life. However, pervasive use of synthetic materials and our excessive dependence on them has environmental consequences. The pressure for sustainable development is shifting the focus from synthetic materials to the utilization of bio-based materials. The same is true for the fiber industry. Carbohydrate polymers, also known as polysaccharides, are the most abundant bio-based, renewable and inherently biodegradable polymeric materials, making up around 75% of all the organic mass on the earth. Hence, polysaccharides provide countless choices and a sustainable supply of starting materials for fiber production. Much effort has been and is being exerted to produce fibers from diverse kinds of polysaccharides. The authors have provided a comprehensive review of polysaccharide fibers that have been fabricated along with a discussion of spinning techniques and fiber applications associated with polysaccharide fibers [1]. In this review we pointed out that, due to the diversity and complexity of polysaccharides, more work on fiber fabrication is required for a thorough understanding of the mechanism and process of fiber formation from polysaccharides.

The carrageenans are linear, sulfated polysaccharides extracted from various species of red seaweed. The “ideal” carrageenan backbone is based on a repeating disaccharide unit of β-D-galactopyranose (A residue) linked through positions 1 and 3, and α-D-galactopyranose (B residue) linked through positions 1 and 4. κ-Carrageenan is one of the three dominant carrageenan species, κ, ι, and λ-carrageenan, which differ in the number and position of sulfate substitutions and presence or absence of a 3,6-anhydride bridge on the B residue. κ-Carrageenan has a sulfate group on the C_4_ of the A residue and has the B residue converted to the 3,6-anhydro form. The presence of this 3,6-anhydride bridge makes κ-Carrageenan helix-compatible and able to gel [2].

**Figure 1:**
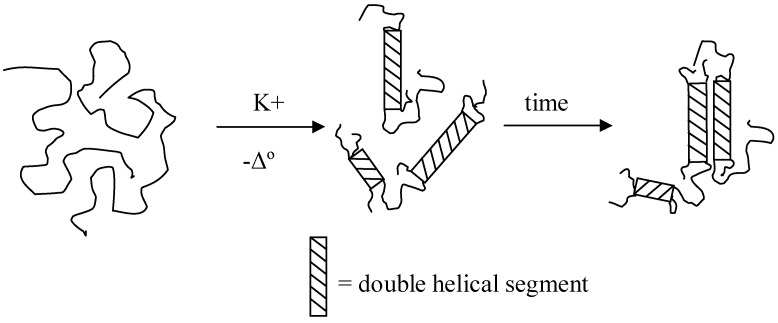
The “two-step” for gelation of κ-carrageenan adapted from [5].

κ-Carrageenan is mainly used in food applications as a texturizing agent, but other applications including cosmetics, pharmaceuticals, and paints are also of importance. The primary utility of κ-carrageenan derives largely from its ability to form cold-setting reversible gels. The gelation process of κ-carrageenan has been extensively studied with respect to the conformational transition of κ-carrageenan molecules [3]. Although the conformational nature, *i.e.*, single or double helices, and their further association during gelation are still under debate, most evidence tends to support the “two-step model” (Figure 1) of coil-helix-gel mechanism proposed by Morris *et al.* [4] as modified by Rochas and Rinaudo [5]. κ-Carrageenan molecules in the sol state adopt a random coil conformation. Upon cooling and in the presence of certain cations, gel network formation from carrageenans proceeds on either a helical or superhelical level. In the first step, each chain joins in double helices with more than one other chain, whereas in the second step, multiple double helices aggregate to form one junction zone of the network.

In addition, the gelling efficiency of κ-carrageenan is cation-dependent. According to Rochas & Rinaudo [6], the helix-stabilizing efficiency of cations was found to follow the sequence Rb^+^ > Cs^+^ > K^+^ > NH_4_^+^ > (CH_3_)_4_N^+^ > Na^+^ > Li^+^ for monovalent cations and Ba^2+^ > Ca^2+^ > Sr^2+^ > Mg^2+^ > Zn^2+^ > Co^2+^ for divalent cations. Picullel [3] thus suggested that cations may be divided into three categories with respect to their helix-promoting efficiency; *i.e.*, the “nonspecific” monovalent cations (Li^+^, Na^+^, (CH_3_)_4_N^+^), the divalent cations (Mg^2+^, Ca^2+^, Ba^2+^, Co^2+^, and Zn^2+^) and the “specific” monovalent cations (NH_4_^+^, K^+^, Cs^+^, Rb^+^). The specific cations are also more effective in promoting helix aggregation than the nonspecific cations. Many properties of the κ-carrageenan gel system, for instance, melting point, elasticity, and yield stress, are tunable due to their sensitivity to the solvent environment. This unique behavior makes the κ-carrageenan gel system versatile and promising for many applications.

The gelation process can be used as an advantage for wet spinning of κ-carrageenan fibers. Wet-spinning is a widely utilized fiber spinning technique, where dissolved material is forced through a submerged spinneret into a coagulation bath containing a non-solvent for the polymer [7]. Generally, the coagulation bath is able to extract solvent from the as-spun fiber and solidify the fiber. Sometimes, ionic exchange (e.g., in calcium alginate fiber production) or chemical reaction (e.g., in the viscose process for cellulose fiber) may occur. Drawing is an optional post-spinning treatment in fiber production and generally beneficial. The consequences of drawing include a decrease in the average fiber diameter, strain induced crystallization and enhanced molecular orientation.

According to our knowledge, this is the first report on fabricating pure κ-carrageenan fibers by a wet-spinning technique. It provides a novel material for many applications, yet will require more research to tailor the fiber properties for specific utilization. Here we present the effects of several spinning parameters, *i.e.*, coagulation bath composition, spinning rate and post-spinning mechanical drawing on the morphological and tensile properties of the resultant fibers.

## 2. Results and Discussion

### 2.1. κ-Carrageenan Characterization

After purification, the κ-carrageenan sample was shown to be much lower in the specific cation (K^+^) as compared with the commercial sample (Table 1). The Ca^2+^ content remained about the same, whereas the Na^+^ concentration increased.

**Table 1:** Cation content of the commercial and purified κ-carrageenan samples, expressed in wt %.

Material	κ-Carrageenan (Gelcarin® GP911 NF)		Purified κ-carrageenan
Method	Data Sheet *	ICP-AES	ICP-AES
K	6.45	4.8	0.68
Ca	1.55	2.4	2.14
Na	0.86	0.72	3.08

* denotes the compositions according to datasheets from commercial providers.

**Figure 2:**
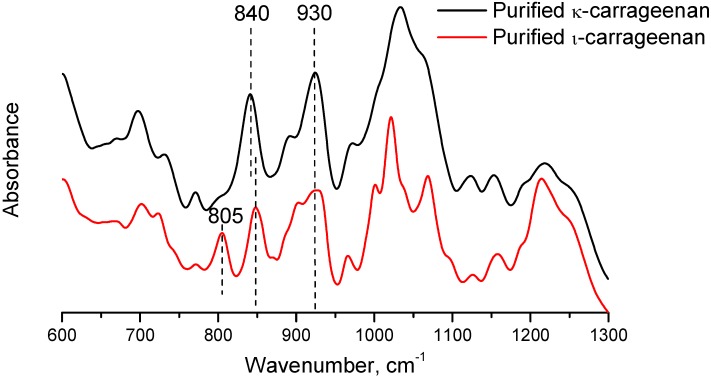
FTIR-ATR spectra of purified κ- and ι-carrageenan samples.

The FTIR-ATR spectra of purified κ- and ι-carrageenan samples are shown in Figure 2. The spectra show two bands at around 840–845 cm^−1^ and 930 cm^−1^, which were assigned to C-O-SO_4_ on the C_4_ of the D-β-D-galactopyranose unit and C-O of the anhydride group on the α-D-galactopyranose unit, respectively [8]. The spectrum of ι-carrageenan also shows a characteristic band at 805 cm^−1^, which is assigned to C-O-SO_4_ on the C_2_ of α-D-galactopyranose residue. This sulfate group is absent in κ-carrageenan backbone. The absence of this band at 805 cm^−1^ suggested that the ι-carrageenan content in the κ-carrageenan sample was negligible.

### 2.2. Effect of Coagulation Bath Composition

The development of a coagulation bath is critical for wet-spinning processes. The coagulation solution may solidify the fiber exiting from the spinneret in different ways. It can provide a cross-linking agent to fix the macromolecules by entanglements. For instance, alginate binds specifically to some divalent cations, e.g., Ca^2+^, in an “egg-box” style gel network, while sodium alginate is generally soluble [9]. As a result, in alginate fiber production, sodium alginate solution is typically used as the spinning dope. This dope is extruded into a coagulation bath containing calcium, where exchange of sodium and calcium ions takes place [10]. Alternatively, the coagulation bath is a non-solvent for the polymer, which can extract solvents from the fiber. This is a widely used tactic in both synthetic fiber production [11] and biopolymer fiber production, e.g., cellulose [12] and silk fibroin [13]. As previously discussed, some specific monovalent cations, e.g., K^+^ and Cs^+^, initiate gel formation in κ-Carrageenan through their effect on the polymer’s net charge; cation binding decreases the net charge of single chain, favoring double helix formation and gelation. Meanwhile, alcohols, e.g., ethanol, are non-solvents for polysaccharides. The combination of these two coagulation mechanisms was thus investigated.

**Figure 3:**
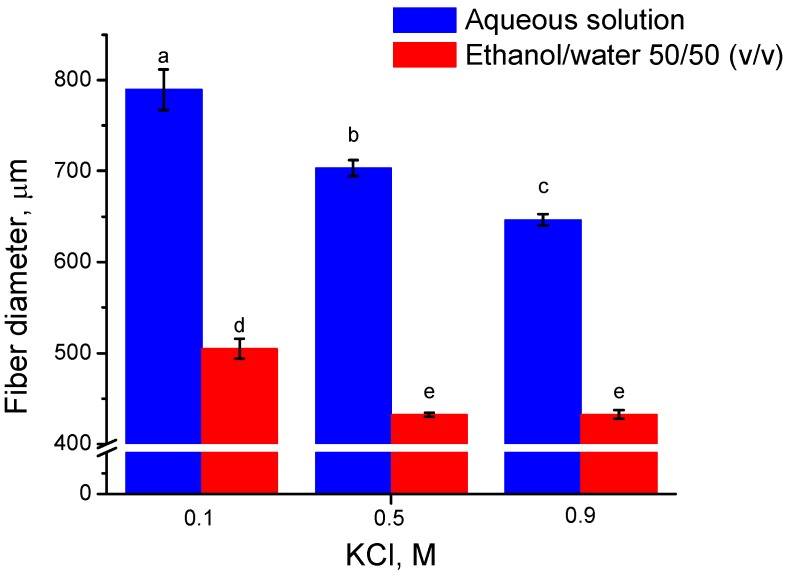
Effect of KCl concentration on as-spun fiber diameters, in either aqueous solution or in ethanol/water (50/50, v/v) mixture (a > b > c > d > e, p < 0.05).

Figure 3 illustrates the effect of coagulation bath composition on as-spun fiber diameter. Different KCl concentrations in aqueous solution and in ethanol/water (50/50, v/v) mixture solution were used as coagulation baths. Given the needle inner diameter 0.51 mm (510 μm), evidence of die swell was prevalent for all coagulation baths without ethanol. The average diameter decreased with increasing KCl concentration. The die swell is the consequence of polymer relaxation to its low entropy conformation after flowing through the needle, where polymer molecules are oriented by the flow. Physical entanglement brought about by K^+^ is thus able to retard the relaxation of κ-carrageenan and retain the oriented conformation of κ-carrageenan in the fiber axis direction. The molecular orientation of κ-carrageenan in the fiber axis direction can be detected by optical microscope with cross polarizers and a quarter wave plate. Higher KCl concentration allows for greater diffusion of K^+^ into the fiber and more rapid phase separation. The addition of 50% ethanol by volume makes the bath a non-solvent for κ-carrageenan and causes the spun κ-carrageenan fiber to collapse. The combined effects from K^+^ and ethanol in the coagulation bath resulted in as-spun fiber diameter of less than 450 µm. The diameter did not decrease further with KCl concentrations of more than 0.5 M in 50% aqueous ethanol.

**Figure 4:**
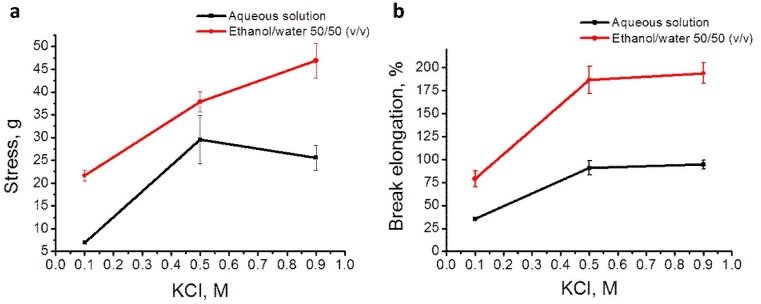
Effect of coagulation bath composition on as-spun fiber tensile properties, ultimate tensile stress (**a**) and elongation at break (**b**).

As-spun wet fibers were subject to tensile tests. Figure 4a and b illustrate the effects of coagulation bath composition on the ultimate stress and elongation at break, respectively. Without addition of ethanol, the ultimate stress and break elongation of the as-spun fiber reached a plateau when KCl concentration reached 0.5 M. The addition of ethanol in the coagulation bath increased both properties. The ultimate stress reached about twice the value without ethanol addition (p < 0.05), and the as-spun fibers could be stretched over two times the length of those without ethanol in the coagulation bath (p < 0.05). The elongation at break also reached a plateau at 0.5 M KCl in the presence of 50% ethanol. It is worth noting that the ultimate stress is expressed as grams in this study, because the as-spun fiber diameter experienced drastic reduction while being stretched. The difference of ultimate stresses with ethanol present or not should be greater if the value is expressed as MPa, taking fiber diameter into account.

**Figure 5:**
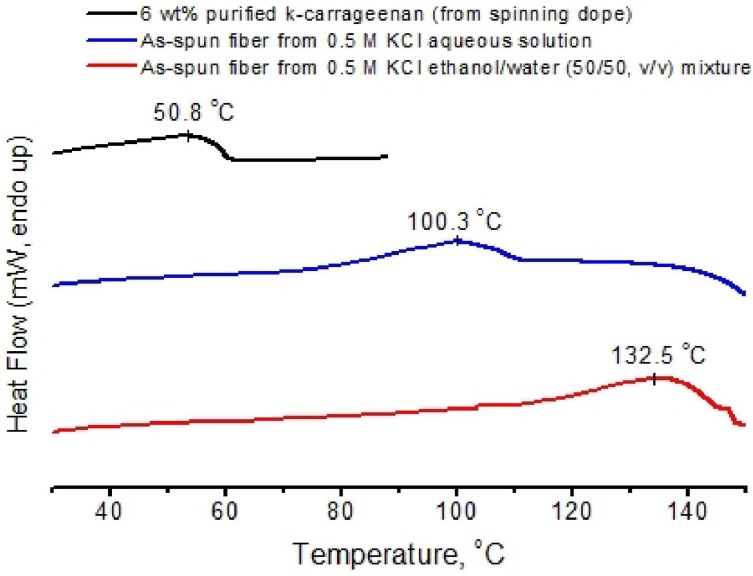
Thermal scans of 6 wt % κ-carrageenan gel and as-spun fibers by differential scanning calorimeter (DSC).

The thermal behavior of as-spun fibers from 0.5 M KCl coagulation bath with and without 50% ethanol was analyzed by differential scanning calorimetry. The results were compared with 6 wt % κ-carrageenan gel (Figure 5). The helix-coil transformation, which is generally assumed to occur coincidentally with the gel-sol transition, of 6 wt % κ-carrageenan gel was broad, with a peak at 51 °C. A fiber with the same polymer concentration showed a similar broad endotherm with a peak at 100 °C. This is likely due to the presence of K^+^ in the fiber which alters the solvent quality and hence the solubility of the κ-carrageenan. The endotherm peak temperature increased to 132.5 °C for the as-spun fiber from 0.5 M KCl in 50% ethanol, since the solvent quality became even poorer.

### 2.3. Effect of Spinning Rate

**Figure 6:**
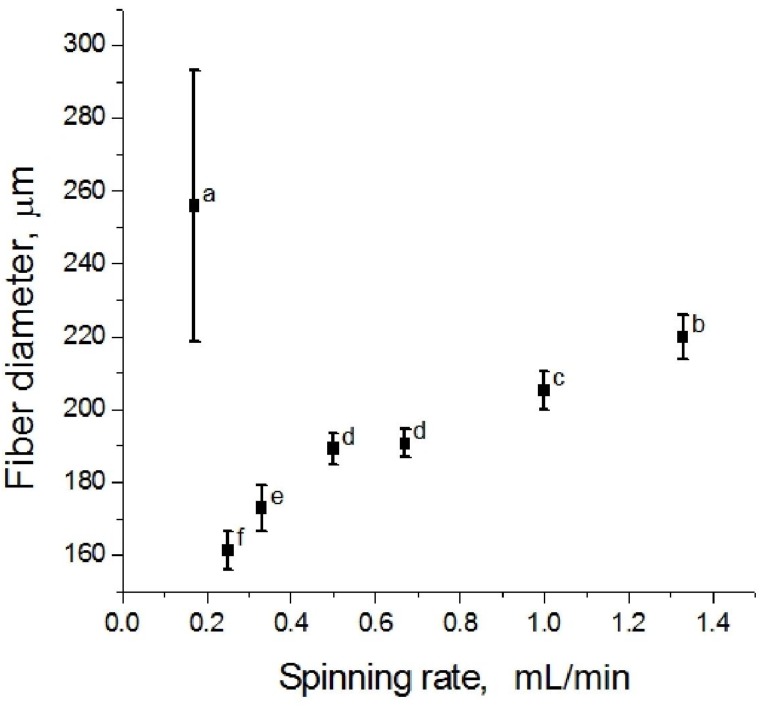
Average fiber diameter as a function of spinning speed (a > b > c > d > e > f, p < 0.05).

**Figure 7:**
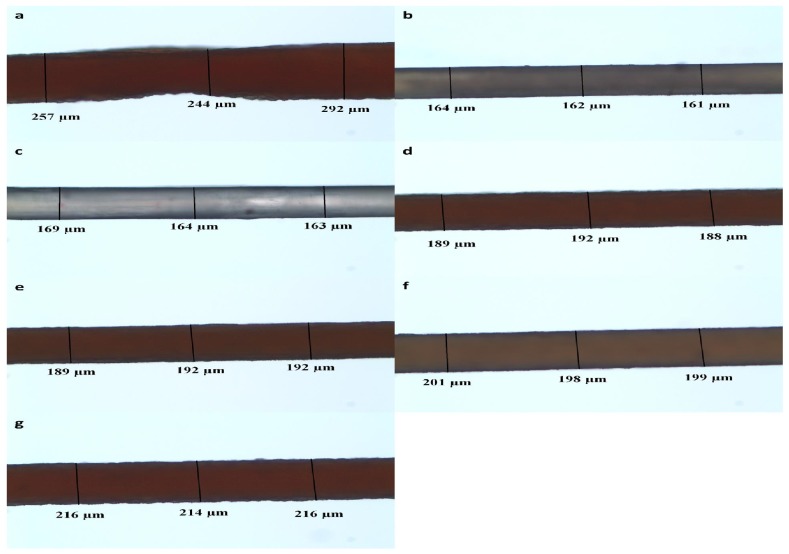
Light micrographs of dried fibers spun at different spinning rates: (**a**) 0.17 mL/min; (**b**) 0.25 mL/min; (**c**) 0.33 mL/min; (**d**) 0.5 mL/min; (**e**) 0.67 mL/min; (**f**) 1 mL/min; and (**g**) 1.33 mL/min.

Fiber spinning rate is another important spinning parameter. Spinning was conducted at 70 °C with spinning rate varying from 0.17 mL/min to 1.33 mL/min. The effect of spinning rate on the diameter and morphology of the dried fibers are shown in Figure 6 and Figure 7, respectively. At the lowest spinning rate (0.17 mL/min), the dried fibers were rough in surface morphology and irregular in cross-sectional shape. The average diameter was 256 µm with a large deviation. It was observed in the experiment that at such low spinning rate, the extruding flow was not smooth but “shish kebob”-shaped. Due to the temperature drop in the flow, the carrageenan dispersion had begun to gel within the spinneret before it reached the coagulation bath. Increasing spinning rate to between 0.25 mL/min and 0.33 mL/min resulted in a smoother fiber surface and smaller average fiber diameters. This suggested that a critical spinning rate is required for the spinning of neat fibers. Further increase in spinning rate gradually increased the roughness of the fiber surface and the dried fiber diameter. The average fiber diameter reached 220 µm at a spinning rate of 1.33 mL/min.

**Figure 8:**
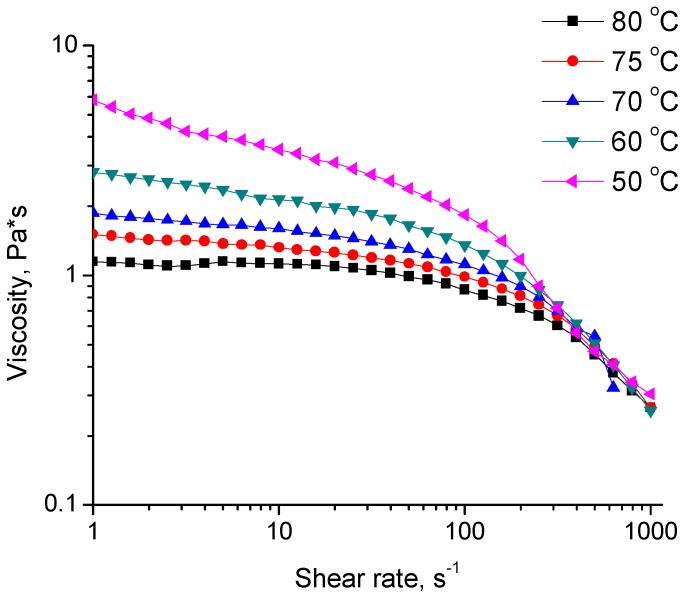
Flow curves of the 6% κ-carrageenan dispersion at different temperatures.

The apparent wall shear rates were calculated to be from 217 s^−1^ to 1702 s^−1^ over the spinning rate range. From the flow curve for the spinning dope (Figure 8), the reader can see that the lowest spinning rate corresponds to a shear rate at the lower edge of the shear thinning region. Hence, this shear rate may be insufficient to fully align the molecules in the flow. At higher spinning speed, shear thinning may have been well developed, where molecules tend to orient in the flow direction and are fixed in the gel network as cation diffuses in. For a given die geometry, the die swell increases with shear rate, which may explain the fiber diameter increase with further increases in spinning rate.

### 2.4. Effect of Post-Spinning Drawing

**Figure 9:**
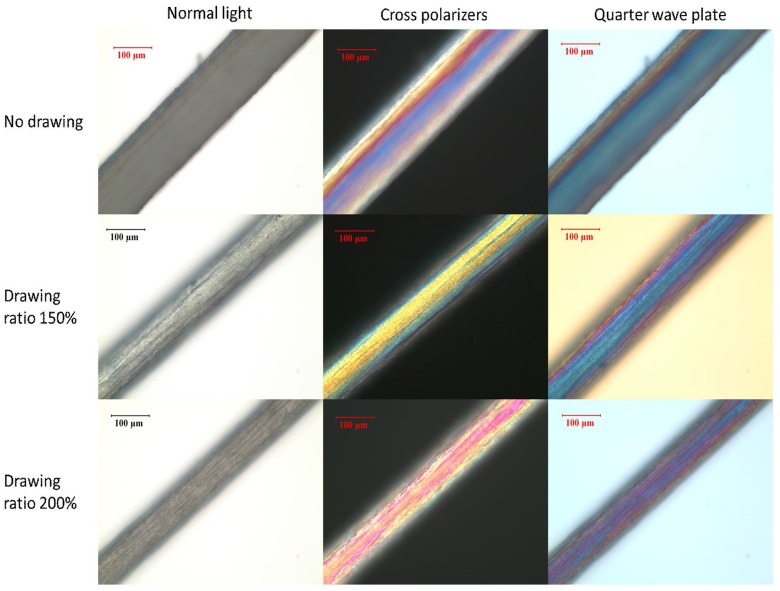
Micrographs of dried κ-carrageenan fibers with and without different ratios of drawing.

We investigated the effect of post-spinning mechanical drawing ratio on the morphology of the κ-carrageenan fibers. Figure 9 shows micrographs of dried fibers produced using different drawing ratios with and without crossed polarizers and a quarter wave plate. Since the drawing speed (0.1 mm/s) was slower than tensile test crosshead speed (0.2 mm/s), drawing ratios as high as 200% could be obtained. The fiber diameter gradually decreased with increasing drawing ratio, reaching about 100 µm at 200% of its original length. The birefringence obtained using crossed polarizers results from orientation of polymer chains in the fiber axis direction. The blue color brought about by the quarter wave plate shows the same molecular orientation. However, the optical micrographs are not quantitative in determining the extent of molecular orientation in the fiber.

## 3. Experimental Section

### 3.1. Materials

κ-Carrageenan (Gelcarin® GP911 NF) was kindly provided by FMC Biopolymers (NJ, USA). Ethanol (200 proof) was obtained from Penn State Chemistry Stockroom. All other reagents were purchased from VWR International and used as received.

### 3.2. κ-Carrageenan Purification

The κ-carrageenan sample was purified from excess salts and low molecular weight carbohydrates by a method modified from Rochas and Rinaudo [6]. In detail, 1.0 wt % of κ-carrageenan was completely dissolved in deionized water heated to 80 °C with stirring. The carrageenan was precipitated with absolute ethanol (4 times the volume of the dispersion). The precipitate was collected using vacuum filtration (Whatman #4, Whatman, Piscataway, NJ, USA). The precipitate was washed in ethanol/water mixture (80/20, v/v) and again filtered. This re-suspension and filtration was repeated 4 times. Finally the precipitate was dried in a forced air oven at 40 °C overnight.

### 3.3. κ-Carrageenan Characterization

The commercial and purified κ-carrageenan was characterized for its cation composition by a Perkin-Elmer Optima 5300 inductively-coupled plasma atomic emission spectroscopy (ICP-AES, Perkin-Elmer, Waltham, MA, USA). Before measurement, samples were dissolved in hot distilled water and acidified. Synthetic standards from High Purity Standards were used to calibrate the results. Fourier transform infrared spectroscopy (FTIR) spectrum of the purified κ-carrageenan samples was recorded using the Bruker IFS 66/S FT-IR Spectrometer (Bruker Optics Inc., Billerica, MA, USA) equipped with attenuated total reflectance (ATR) accessary containing diamond crystal. The spectra were scanned at room temperature over the wave number range of 4000 to 400 cm^−1^, with an accumulation of 400 scans and a resolution of 8 cm^-1^.

### 3.4. Wet-Spinning

Spinning dope was prepared by dissolving purified κ-carrageenan in deionized water at 80 °C for at least 1 hour. The dispersion was homogeneous by visual observation before spinning. A dope concentration of 6 wt % was used throughout the study. Wet-spinning was carried out using a bench-top device. A jacket-type circulating device (Penn State Glass Shop) was used to maintain the spinning dope temperature in a 3 ml syringe (Becton, Dickinson and Company, Franklin Lakes, NJ, USA). The dope was extruded by a syringe pump (Cole-Parmer 74900, Vernon Hills, IL, USA) through a blunt stainless steel needle (20 G, 0.51 mm) into a coagulation bath. The as-spun fiber was kept in the bath for at least 2 hours to ensure complete ion diffusion into the fiber.

### 3.5. Morphological Characterization

Observation of fibers was performed using an Olympus BX41 optical microscope (Hitech Instruments, Edgemont, PA, USA) equipped with cross polarizers and a SPOT Insight QE camera (SPOT Diagnostic Instruments, Sterling Heights, MI, USA). Analysis was completed using SPOT analytical and controlling software.

### 3.6. Thermal Characterization

Thermograms were recorded using a using a differential scanning calorimeter (DSC Q100, TA Instrument, New Castle, DE, USA), and 60 µL hermetic stainless steel pans (Perkin-Elmer Instruments, Bridgeville, PA, USA). For scanning of gels, at least 50 mg of the spinning dope was filled into the pan. For scanning of fibers, at least 30 mg of as-spun wet fibers were loaded into the pan without additional solvent. Samples were equilibrated at 10 °C, then heated to 170 °C at a scanning rate of 2 °C/min. The DSC was calibrated with indium, and an empty sample pan was used as a reference.

### 3.7. Rheology

Flow curves were generated using parallel plate geometry on a strain-controlled rheometer (ARES, TA Instrument, New Castle, DE, USA). The parallel plate diameter was 50 mm and the gap was set at 0.5 mm. Sample was loaded between the plates when hot and sealed by mineral oil on the periphery to avoid solvent evaporation.

### 3.8. Tensile Test and Drawing

Fiber tensile tests and drawing were performed on a Texture Analyzer (TAXT2i, Stable Microsystems, Godalming, UK) with film/fiber clamps. The clamp sawteeth were covered with a piece of foam and a piece of paper to avoid slip and fracture of fibers at the clamp sawteeth. Initial fiber length was 20 mm. Crosshead speed was set at 0.2 mm/s for tensile test and 0.1 mm/s for drawing. The ultimate tensile stress (g) is the maximum stress applied to break a single fiber. The break at elongation (%) is defined as the ratio of the elongated length at break to the initial fiber length (20 mm).

### 3.9. Statistical Evaluation

For the measurements of fiber diameters, at least 10 spots on the fibers were measured and averaged. For tensile tests, 3 fiber samples were examined. Quantitative data were presented as mean ± standard deviation (SD). Student’s t-test and one-way ANOVA were conducted. p < 0.05 indicated significant difference.

## 4. Conclusions

In the present study, we investigated the effect of coagulation bath composition, spinning rate and post-spinning mechanical drawing on the morphology and in some cases tensile properties of κ-carrageenan fibers produced by a wet-spinning technique. The as-spun fiber diameter decreased with KCl and ethanol concentration in the coagulation bath. In general, the ultimate tensile stress and elongation at break both increased for KCl concentrations from 0.1 M to 0.5 M with and without ethanol, and enter a plateau region thereafter. We suggest that the improvement in tensile properties resulted from more K^+^ diffusion into the fiber and fiber collapse brought about by ethanol addition. Spinning rate affected the dope flow and thus the polymer orientation (apparent viscosity) and fiber morphology. At spinning rates of between 0.25 ml/min and 0.33 ml/min, the fiber diameter reached a minimum and the fiber surface was smooth. Both an increase and a decrease in this spinning rate range increased the fiber diameter and roughness of the fiber surface. Post-spinning drawing of the fiber resulted in even smaller fiber diameter.

This is the first time that wet-spinning of pure κ-carrageenan fibers have been discussed. The success of spinning κ-carrageenan fibers provides new materials for food, textile, filtration and biomedical applications.
